# The Kantian background of Uexküll’s notions of time and space

**DOI:** 10.1007/s40656-026-00722-9

**Published:** 2026-03-19

**Authors:** Jonas Müller

**Affiliations:** Radboud University Nijmegen, Nijmegen, Kingdom of the Netherlands

**Keywords:** Jakob von Uexküll, time, space, Immanuel Kant

## Abstract

The Baltic-German biologist Jakob von Uexküll was heavily inspired by the work of philosopher Immanuel Kant. Uexküll’s views on time and space were likewise inspired by Kant. Kant argued that time and space are the a priori forms of intuition. Uexküll argued similarly that time and space depend entirely on the subject. It is the subject’s biological constitution that brings forth time and space, and this conceptual pair is therefore a foundational part of the subject’s world. But Uexküll also took inspiration from other authors. The aim of this paper is to investigate how Uexküll adapted Kant’s notions of time and space and how this adaptation is related to another investigation into the topic of time and space by the biologist Felix Gross. To achieve this, I examine how Gross’s work shaped Uexküll’s adaptation of Kant’s ideas. I show how Gross’s essay on Kant’s views on time and space shaped Uexküll’s alteration and naturalisation of Kant’s pure forms of intuition.

## Introduction

Jakob von Uexküll (1864–1944) was a Baltic-German biologist and physiologist whose work has found reception among many influential philosophers of the twentieth century.^1^ More recently, researchers in the field of biosemiotics, the study of meaning-making in the biological realm, have turned to Uexküll’s work for inspiration.^2^ Indeed, Uexküll is seen as an inadvertent founding father of the field (Brentari, 2015, p. 2; Magnus & Kull, 2009, p. 125). The rich philosophical background of Uexküll’s work, however, is not as well known.^3^ Therefore, a closer examination of the sources that shaped Uexküll’s own thinking is warranted.

Two of the main influences on Uexküll are the works of the well-known philosopher Immanuel Kant on the one hand, and the neovitalist tradition on the other hand. Suffice it to say that Kant argued that the subject actively shapes its knowledge of objects through its mental apparatus. That is to say that we do not simply passively perceive the world around us, but rather that our minds construct this world. Nevertheless, Kant also argued that there is a world that exists independently of the subject, which is the thing-in-itself. We can however only know that there must be a world in itself. We cannot know anything more about it, since we can never step out of ourselves to see the world as it really is independently of the subject. Neovitalism, the other main influence on Uexküll’s work, is characterised by the denial that life could be explained by mere mechanistic and physical processes. Hence, neovitalist thinkers reserved space in biology for final causes and immaterial vital forces that bestow upon organisms whatever it is that makes it living.^4^

Uexküll came to use Kant’s view of the subject as outlined above for his well-known concept of the *Umwelt*. Put briefly, *Umwelt* refers to the subjective environment that each animal constructs through its constitution. The *Umwelt* is composed of two parts: the *Merkwelt* and the *Wirkwelt*. The *Merkwelt*, which can be translated roughly into perception world, is the world that is perceived by the animal subject. The *Wirkwelt*, which might be called the effect world, is the world the animal acts upon (Uexküll & Kriszat, 1956, p. 22).^5^ The animal subject is entirely enclosed in this *Umwelt*, and its *Umwelt* is closed off to other subjects (Uexküll & Kriszat, 1956, p. 109).

In his 2015 monograph on Uexküll, Brentari notes that time and space gain central importance in the biologist’s 1920 work *Theoretische Biologie* (2015, p. 139). In fact, Uexküll dedicates the first two chapters of that work to the topic of time and space. This attests to the central importance of time and space for Uexküll’s system, and he would come to refer to them as foundational qualities [*Grundqualitäten*] of the *Umwelt* (1928, pp. 58–59). Uexküll fundamentally agrees with Kant’s assessment. Time and space serve as the necessary background for the possibility of the subject’s experience. But Uexküll saw his work as going beyond Kant (1928, p. 44). To achieve this, he drew on the work of another biologist who is much less known today, Felix Gross.

The aim of this paper is to investigate how Uexküll adapted Kant’s notions of time and space and how this adaptation is related to Gross’s investigation into the topic of time and space. To achieve this, I will firstly provide a brief overview of relevant ideas from the work of Kant in § 2. In § 3, I will offer an explication of Uexküll’s concept of time and space. Since Uexküll’s first programmatic discussion of both time and space appears in his *Theoretische Biologie*, my analysis will focus mostly on that particular work, although occasional excursions will be made into his popular 1934 *Streifzüge durch die Umwelten von Menschen und Tieren* (henceforth *Streifzüge*).^6^ Finally, I will examine how the work of Gross shaped Uexküll’s adaptation of Kant in § 4. I will show how Gross’s essay on Kant’s views on time and space would shape Uexküll’s alteration and naturalisation of the pure forms of intuition.

## Kant

### Kant on time and space

As in most other areas of Uexküll’s philosophical biology, Kant’s influence on the biologist’s ideas on time and space cannot be overlooked. In short, Kant held that space and time are pure forms of sensible intuitions (B35-6).^7^ That is to say that they are necessary preconditions of experience. Time and space themselves are not given in experience but rather precede them. Space is of course a necessary ingredient in our experience, because one perceives things and events that take place in space. Time is likewise needed to provide the sequential order of the experience. Yet we do not perceive time and space themselves with our senses. Hence, time and space must be given a priori to our mental capacities in order to structure our experience.

Kant provides a couple of related arguments that space and time are separate from the events that take place in them. Firstly, space and time are needed as the basis of our perception. They do not derive from perceptual experience, but must underlie it. Secondly, I can image that there are no objects in this room whatsoever. What I cannot imagine, however, is there being no space whatsoever. Likewise, I cannot remove time from any appearance, but I can easily imagine time without any appearance. Hence, space and time can be separated from the objects and events that take place in them (B 39; B 46 − 7).

### Kant on apperception

A central concept in the work of Uexküll is apperception. As will become clear in § 4.2, Uexküll generally used apperception as a catch-all term for the synthetic powers of the subject. But before an analysis of Uexküll’s use of the term can be given, a brief overview of the concept as it was used by Kant is needed. Kant distinguished between empirical apperception and transcendental or pure apperception. Empirical apperception is the apprehension of one’s mental states. Kant defined it as follows:

Consciousness of oneself according to the determinations of our state in inner perception is merely empirical and always transient. There can be no fixed or permanent self in this stream of inner appearances. It is usually called inner sense, or empirical apperception. (A107)

In layperson’s terms, empirical apperception is basically the ability to observe what we might now call a stream of consciousness. The contents one apperceives are constantly changing. When I reflect on what I am experiencing now, for example, I become aware that I am experiencing ever-changing perceptions: I see colours here, hear loud sounds over there, feel a draft now, and warmth later. This is the “flow of inner appearances”, in which nothing enduring like a self can be found.

However, Kant argued that there has to be an “I” or self that organises its experiential data into a unity. Kant called this the “pure, original and unchangeable consciousness” (A108). This “I” is not given empirically, but is rather the precondition for any knowledge whatsoever: “No knowledge can take place in us … without that unity of consciousness that precedes all data of intuitions” (A108). This Kant called the pure or transcendental apperception.

Kant then makes the distinction between the analytic and the synthetic unity of apperception. On the analytic unity of apperception, Kant wrote that the “principle of the necessary unity of apperception is itself an identical and therefore an analytic proposition” (B135). The idea seems to be that what must be common to all my representations is that they belong to the same I: “[T]he *I think* is itself the thought of what is common to all conceptualization” (Allison, 2004, p. 172). That is to say that the *I think* is the formal requirement for any representation whatsoever. If this were not present, I could obviously not identify different representations as belonging to the same consciousness. So any representation I have must belong to the same consciousness.

However, the analytic unity of apperception “presupposes, as a condition of its possibility, a synthetic unity of apperception, which somehow combines all the representations the *I think* must be able to accompany” (Engstrom, 2013, p. 37). To make sense of this, a brief summary of what Kant meant by synthesis is needed: “By synthesis … I mean the act of putting different representations together, and of comprehending their manifoldness in one item of knowledge” (B103). I might, for example, have some sensations of something that is hard, more or less spherical, red, sweet, and so on. Through the act of synthesis, this manifold is composed into a single item of knowledge. So all the properties just described are combined to form a representation of an apple.

On the synthetic unity of apperception, Kant wrote:

Only because I am able to combine a manifold of given representations in one consciousness is it possible for me to represent to myself the identity of the consciousness in these representations, that is, only under the presupposition of some synthetic unity of apperception is the analytic unity of apperception possible. (B134)

In other words, the analytic unity of apperception is entirely dependent on the synthetic unity of apperception. It must be possible for me to synthesise any representation in the first place in a single consciousness. Otherwise, I would not even be able to recognise it as being *my* representation, but would have “as many-coloured and varied a self as I have representations of which I am conscious” (B134).

After this summary examination of a few central concepts of Kant’s philosophy, it will be possible to examine Uexküll’s views on time and space and to investigate Gross’s work and the biologists’ use of the concept of apperception.

##  Uexküll on time

### Time and number in *Theoretische Biologie*

Although fundamentally in agreement with Kant’s views on time as outlined above, Uexküll added his own analysis which he saw as expanding on Kant’s work.^8^ Uexküll argued that time consists of moment-signs [*Momentzeichen*]. These moment-signs are the smallest units of time we can experience (Uexküll, 1928, p. 45). In *Streifzüge*, these moment-signs are referred to as elementary sensations [*Elementarempfindungen*] (Uexküll & Kriszat, 1956, p. 46). However, the moment-signs in themselves are not yet enough to constitute our experience of time. They are rather like empty forms, which are filled with qualities, like sensations of mental or external events. The moment-sign and the quality that fills it together produce an experienced moment [*Moment*] (Uexküll, 1928, p. 45). The moment-signs are thus rather like a roll of unused film. Before the film is used, each frame itself shows nothing. Only when the film is properly used in a camera, events can be captured on it. So whereas the blank, unused frames of a film are like the moment-signs, the used film’s frames are like the moments. Time, in short, is nothing more than a chain of moment-signs. We experience events, whether they be mental or external, temporally because the moment-signs are receptive to being filled in with sensations.

Indeed, the analogy between time and film is used by Uexküll himself when he turns to the times of different subjects (Uexküll & Kriszat, 1956, p. 47). Each subject produces its own time, which means that there are as many times as there are subjects (Uexküll & Kriszat, 1956, pp. 46–47). That is to say that, because each subject is constituted differently, each subject will possess different amount of moment-signs. For example, the more moment-signs a subject has, the longer its time is and vice versa (Uexküll, 1928, p. 51). This can be explained easily when one returns to the film analogy. A subject that possesses many moment-signs will experience time slower, rather like the film technique that is referred to as slow-motion. However, time will appear much faster to a subject who has relatively fewer moment-signs. A suitable analogy here might be time lapse photography, which render processes that appear slow to us under normal circumstances as developing much quicker.

But how do we determine such facts about another subject’s Umwelt? That can be achieved through experiments (Uexküll & Kriszat, 1956, pp. 47–48). Uexküll referred to an experiment where a snail is fixed on a rubber ball so that it can crawl yet stay stationary. A stick is then used to tap the snail. If the snail is tapped by the stick three times a second, the animal will recoil. Yet if the animal is tapped four times a second or more, it will attempt to crawl on the stick. The conclusion Uexküll drew is that the stick that moves at that rate appears in the snail’s *Umwelt* as a solid object, which is why the snail tries to crawl on it (Uexküll & Kriszat, 1956, p. 48). It follows that the snail is capable of experiencing about four moments a second, which in terms of the film analogy means that it has fewer ‘frames’ than a human subject has. This in turn implies that compared to our perception, the snail experiences everything as moving much faster, in much the same way time-lapse photography can be used to show processes that appear slow to us as being much faster.

The different means and instruments we humans use to measure time might persuade someone to conclude that there must be an objective time after all. Indeed, do we not measure time as it is in itself with our pendulums and watches and atomic clocks? Uexküll, however, rejects this notion (1928, p. 46). The objects that can be used to measure time do not point to some entirely objective, that is to say subject-independent, time. Objective time is nothing more than a convention we use to get along with each other more easily (Uexküll & Kriszat, 1956, p. 46). It is a very useful idea in both our everyday lives and our scientific endeavours, but that does not mean that objective time really exists. We must not think that a clock measures anything external to the subject.

But the question of the relationship between objects we use to measure time and time itself remains. If there is no objective time that exists independently of subjects, what then is it that we measure with our clocks? As seen, time is composed of little atomic time slices called moment-signs. When we measure the duration of an event, what we are actually attempting to measure is the amount of moment-signs in which that event took place (Uexküll, 1928, p. 45).

Of course, we measure these moment-signs with number. Therefore, Uexküll thought an exposition on the nature of number was in order at this point. Basically, Uexküll considers number to be an artefact [*Kunstproduct*] (1928, p. 46 − 47). That is to say, the use of numbers likely developed out of human’s ability to produce a beat or rhythm [*Taktschlagen*] (Uexküll, 1928, p. 46). By producing a repetitive beat, as with a metronome, some moment-signs are so to speak filled in with a sound. This helps us to distinguish moment-signs from one another in a relatively reliable way, since some are filled with the rhythmic sound, while others are not. Due to the regularity of the sound, it can be used as an approximate measure of the moment-signs and hence of time.

It seems that Uexküll, who was particularly fond of musical metaphors and analogies, argued that some basic elements of musicianship enabled human subjects to devise the concept of number.^9^ An example will be useful at this point. Say a subject has five moment-signs, call them A, B, C, D, and E. Without a regular measure, it can be difficult to distinguish these moment-signs from each other. But say there is such a measure, like a metronome. Of course, if the metronome’s ticking filled all five moment-signs, we would experience it as a single continuous sound. In that case, the sound could not be used to distinguish between the moment-signs. But if the ticking of the instrument fills moment-sings A, C, and E, whereas B and D remain empty. The subject can then use the rhythmic ticking of the metronome as a measure of time and conclude that five moment-signs have passed. For the subject experiences three moment-signs that are filled, and two moment-signs that are not filled with the sound of the metronome.

Uexküll then continued by speculating that a primitive form of numbers came about by humans drawing lines in the sand with a finger on the basis of a beat or rhythm (1928, p. 47). We externalise this by drawing lines in the sand, which is the origin of number. Since the externalisation of this counting takes place in space, number ties together the measurements of time and space (Uexküll, 1928, p. 47). After all, just as time can be measured by a regular but interrupted sound that fills some moment-signs and leaves others empty, space can be measured by a regular visual cue that fills some spaces yet leaves others empty (Uexküll, 1928, p. 48). One might think of a ruler, which has black stripes interrupted by white spaces so that the user can measure a certain magnitude of space.

Before continuing, a critical note must be made. In his approach to measuring the times of other subjects, I believe, Uexküll’s account of time encounters a major difficulty. If there is no objective time beyond what the subject constructs, how can we ever measure the time of other subjects? All we can do is measure their responses and behaviour with the help of our own temporal measurements. In the experiment of the snail, for example, how do we say that a snail experiences time faster if there is no universal unit of measurement? All Uexküll could possibly conclude is that the time of the snail moves faster relative to our human time.

The problem is even more dire when one takes his Kantian background into account. As seen earlier, Uexküll argued that the *Umwelten* of other subjects are strictly speaking off-limits for us. As Kant said, after all, the subject cannot, so to speak, step outside of itself. Rather, the subject can necessarily only know what is constituted by itself through its mental apparatus. Yet Uexküll apparently saw no contradiction in applying the laws of our human time to non-human subjects, as he does in the example with the snail. What is his justification for doing this? I will return to this difficulty in § 3.3.

### *Merkzeit* and *Wirkzeit*

Only perception time [*Merkzeit*] has been discussed so far, which concerns our apperception according to Uexküll (1928, p. 55).^10^ That is to say that it is the time in which the subject experiences phenomena that take place outside of it in the world around the subject, or within it, like memories of sensations and other mental states. But as discussed in the introduction, the *Umwelt* is co of a *Merkwelt* and *Wirkwelt*. Likewise, there exists not only a perception-time [*Merkzeit*], but also an operative time [*Wirkzeit*]. Operative time can be best summarized as the time of a subject’s muscle contractions (Uexküll, 1928, pp. 55–56). To simplify, perception-time concerns the time it takes to perceive something, but operative time is the time to do something.

As described in § 3.1, Uexküll had established that the shortest unit of perception-time is the moment. Likewise, operative time has such a smallest unit. This smallest unit must refer to the shortest possible muscle activity, like blinking is in humans (Uexküll, 1928, p. 56). To find a suitable term to refer to this smallest unit, Uexküll takes recourse to the French language. He considers “un chin d’oeil”, which means something like “a blink of an eye”, to be a suitable candidate. Seemingly not content with the German word “Augenblick”, which means “blink of an eye”, Uexküll proposes to use the “good old” German word “Nu” (1928, p. 56). Unlike the moment, the *Nu* has not yet been measured (Uexküll, 1928, p. 56). But whatever duration the *Nu* has, it must concern the smallest muscle activity.

Uexküll’s example of the housefly’s perception time and operative time is useful here (1928, p. 55). According to Uexküll, the perception time of the fly passes rather slowly. But the fly’s reaction time is quick, because the interval between the operation of the antagonistic muscles in its wings is very brief. In other words, the time between the operation of the muscles that push and pull the fly’s wings is short, which allows the fly to act quickly. Presumably, then, the insect’s *Nu* is much smaller than ours, which is why it can move around so much faster than we humans can.

As is the case with perception time, operative time can also be measured. However, perception time and operative time have fundamentally different sources. Although Uexküll does not go into detail about how perception time is created, it is related to apperception, and is hence a product of the *Gemüt* (Uexküll, 1928, p. 55).^11^*Gemüt*, as Uexküll uses it, refers to the totality of mental capacities (1928, p 3).^12^ Somehow, then, the mind organizes perception temporally. However, operative time is a product of something else which is, in the words of Uexküll, built-in to our mental life (1928, p. 55). This something else are the impulses of the will [*Willensimpulse*] (Uexküll, 1928, p. 55). Earlier in the *Theoretische Biologie*, Uexküll had posited that the actions of the subject are ultimately derived from some unknowable source (1928, pp. 21–22).^13^ This source sends orders, the impulses of the will, to a subject’s muscles, which then puts the subject in motion.

The sources’ commands or directives [*Willensdirektive*] are temporally ordered (1928, p. 22). After all, one impulse follows the other. However, their source can only be known to us indirectly:

Here we come up against a natural factor that acts within us which remains unknowable, despite it being fitted into our mind [*Gemüt*] in a plan-like fashion … From a source that is inaccessible to us, the temporally ordered chain of directives of the will uncoils. (Uexküll, 1928, p. 22)^14^

In summary, an unknowable source sends out directives to the subject, which the muscles then obey. These directives are temporally ordered, and hence this is what operative time must ultimately refer to. When it comes to measuring the *Nu*, however, it becomes less clear how one should achieve that. Uexküll notes that the *Nu* has not been measured yet, and it may be the case that by measuring the muscle activity, all work is basically concluded: “The result may be that the total timespan of the *Nu* can be entirely ascribed to the activity of the muscles and nothing remains for the impulse of the will” (Uexküll, 1928, p. 56).^15^ It appears then that Uexküll is suggesting that perhaps only muscle activity can be measured, but the how remains unexplored.

### The thing-in-itself

In his discussion of the source of the impulses of the will, Uexküll’s vitalistic interpretation of Kant’s thing-in-itself becomes apparent. This might offer a solution to the problem outlined in § 3.1, namely that Uexküll makes conclusions about the *Umwelten* of other subjects. Nature is to be regarded as a harmonious whole in which everything is structured according to a plan [*planmäßig*] (Uexküll, 1928, p. vi). Some immaterial principle or principles organise nature, like the mysterious source of the impulses. This principle belongs to the thing-in-itself, as something that determines the world of appearances but cannot be known directly by the subject.

However, since everything in nature is ‘in tune’ owing to the immaterial principles at play, subjects are actually not entirely closed off from each other. This harmony and lawlikeness of nature guarantees that the *Umwelt* of different subjects are not too dissimilar (Uexküll, 1928, p. 68). In another work, Uexküll mentions the spider’s web as an example:

Surely, the spiders web is configured in a fly-like way, because the spider is also fly-like. To be fly-like means that the spider has taken up certain elements of the fly in its constitution … Better expressed, the fly-likeness of the spider means that it has taken up certain motifs of the fly melody in its bodily composition. (Uexküll & Kriszat, 1940/2010, pp. 190–191)

In other words, the spider must have something that is ‘fly-like’ in its constitution. If this were not the case, the spider could not hunt flies, since the fly would then be completely non-existent in the spider’s *Umwelt*. It follows that we humans too have something in our bodily composition which resembles other animal subjects, which guarantees that we can ascertain certain details of those subject’s *Umwelten*, albeit only indirectly through estimated guesses on the basis of empirical research. Only to our mind’s eye can the *Umwelt* of another subject become visible (Uexküll & Kriszat, 1956, p. 22). Hence, we are justified in our analysis of the snail’s time, since, as odd as it may sound, humans have something snail-like in our constitution.

The concern remains, of course, that appealing to an immaterial force is again going against the Kantian proviso that the subject ought to refrain from making claims about the thing-in-itself. All one might say is that there is a thing-in-itself, independent of the subject. Any positive claims about this thing-in-itself already go beyond the capacities of the subject. Hence, to argue that some immaterial force makes possible our research into the laws is not a satisfying solution from a Kantian perspective. A thorough discussion on the incompatibility between Kant’s philosophy and Uexküll’s vitalism is beyond the scope of this paper’s aims. Suffice it to say that this tension between vitalist and Kantian influences is a motif in Uexküll’s work that was never fully resolved.^16^

### Time, space, and motion

There is a common view in the history of philosophy that space and time are closely related concepts. Notably, it was argued that there exists considerable symmetry between the qualities of space and time. Ezio Vailati has referred to this as the “parity between space and time” (Vailati, 1998, p. xviii). That is to say that what goes for space, is generally also the case for time and vice versa. Time and space are a conceptual pair, closely related and possessing similar properties. Take, for example, Kant’s discussion on time and space as described above. His arguments for time and space being pure forms of intuition are nearly identical by virtue of the shared properties of time and space.

The parity between time and space is also present in Uexküll’s work. Time and space together are the foundational properties [*Grundqualitäten*] of the subject’s *Umwelt* and together influence the third, which is motion (Uexküll, 1928, pp. 58–59). But to understand this, a brief overview of the basic elements Uexküll’s concept of space is needed. Just like time, Uexküll bifurcates space into perception space [*Merkraum*] and operative space [*Wirkraum*] (Uexküll, 1928, p. 15). The former is, of course, the space which the subject perceives, while the latter is the space in which the subject acts.

Uexküll considered space, like time, the product of the subject’s constitution (1928, p. 4). A subject’s space ultimately depends entirely on certain parts of its body. In vertebrates, it is the semicircular canals that guarantee that we experience the three dimensions of space (1928, pp. 18–19; Uexküll & Kriszat, 1956, pp. 32–34). Animal subjects that lack this organ will not experience space the same way. Uexküll claimed that the relation between the semicircular canals and space were demonstrated by Elias von Cyon, a Russian-French physiologist who did extensive research on said organ (von Cyon, 1908).^17^ Either way, as space depends entirely on the constitution of the subject, it is entirely subjective. There is no such thing as an objective space that is independent of the subject, just as there is no objective time.

As described above, time is composed of smallest bits, the moment-signs. Likewise, space is composed of atomic bits. These smallest parts Uexküll calls local-signs [*Lokalzeichten*] (1928, p. 5). The cones and rods in the eyes determine the amount of these local-signs in visual space, whereas the nerve endings in the skin determine the amount in tactile space (Uexküll, 1928, pp. 8–10). If moment-signs are like an empty roll of film in which events are ordered sequentially, local-signs are like an empty grid in which events and objects are ordered spatially. The more cones and rods the eye possesses, the finer this grid becomes. But like the moment-signs, these local-signs themselves are empty. Much like how an empty sheet of grid paper can be filled with drawings, the local-signs can be filled with sensations. When the local-signs are filled with qualities, however, they become a place [*Ort*] (Uexküll, 1928, p. 24).

The third foundational property of the *Umwelt* is motion. Naturally, motion requires both time and space, since it takes place over a given amount of time and space (Uexküll, 1928, pp. 52–53). The directly proportional relation between moments and places mean that we can use them to calculate how motion appears in another subject’s *Umwelt* (Uexküll, 1928, pp. 54–55).^18^ In short, motion increases when the places per moments are many, but it decreases when there are many moments per places (Uexküll, 1928, p. 55). Consider a motion that occurs over three places, but only in one moment. To this subject, this motion will appear quick, because it traverses three places in only one moment. But now imagine the same motion over three places, but a certain subject perceives this in three moments. To this subject, the motion will appear relatively slow, because it takes three moments instead of just one to complete.

Imagine that we somehow gained thousands of receptors in our eyes. The consequence would be that we could distinguish between many more places than we can do now (Uexküll, 1928, pp. 60–61). But this would be pointless for us, since if our moments stayed the same, motion would become too fast for us. A snail would then appear to move as fast as a horse, since it would travel many more places in the same amount of time. If the moments however increased relative to the *Orte*, every event would appear stretched out. In this case, everyday life would become too long and exhausting for humans. Thankfully, our moments and places are exactly right for our biological needs. This is because every subject is perfectly fitted into its *Umwelt*. Therefore, humans, and all other subjects, have just the right amount of places and moments.

It should lastly be noted that for Uexküll, time and space are not rigid. Although it is true that elements of the *Bauplan* determine their basic properties, certain other properties of time and space can nevertheless be altered. Certainly humans can learn to ‘see’ new spaces. Our experience of space is not something that is entirely innate and unchanging. Rather, it is something we develop over our lifetimes. In our visual space, we have something which Uexküll refers to as the horizon or the farthest plane [*fernste Ebene*] (Uexküll & Kriszat, 1956, p. 42). When we look at the starry night sky, for example, it appears as a final barrier of our visual space. We cannot look behind that backdrop which surrounds all that is visible. But the farthest plane can shift, and generally we learn to move this farthest plane away farther and farther as we get older (Uexküll & Kriszat, 1956, pp. 42–44).

Uexküll used an anecdote of a child learning about perspective to illustrate this (Uexküll & Kriszat, 1956, pp. 42–44). During a walk with his mother, a young boy sees workmen walking around on a church. The boy’s farthest plane is still relatively close. Hence, to the boy, the church does not appear to be far away, but close and small. Therefore, the boy asks his mother if she could grab the little dolls from the building and give them to him. The mother, however, sees the church not as small and nearby, but large and far away. That is because her farthest plane is much farther away than her son’s. In the *Umwelt* of the boy, the church is nearby but small. In the *Umwelt* of the mother, the church is large but far away.

In regard to time, as discussed previously we can use technologies like time lapse photography or slow motion to render motions visible to us which were previously invisible (Uexküll & Kriszat, 1956, p. 47). With such techniques, we either stretch out the specific motion over a longer period of time, or quite the opposite, make the motion take place in a shorter timeframe. But Uexküll apparently did not hold that our time experience itself could develop in the way our experience of space can. So whereas we do actually expand space when we learn to see perspective, we do not do the same for time.

## Felix Gross and Jakob von Uexküll

### Felix Gross

Gross is mentioned by Uexküll as another influence on his work (1928, p. 44). A closer inspection of Gross’s work reveals a strikingly similar project to Uexküll’s, both of which in effect naturalised Kant’s views on time and space. Very little is known about the life of Gross, other than that he was a biologist born in Vienna in 1889 (Mildenberger, 2007, p. 95). He published a treatise on space- and time-perception in 1910 called *“Form” und “Materie des Erkennens in der transzendentalen Ästhetik*, in which he critically engaged with Kant’s transcendental aesthetic.

As described above, Kant argued that input of time and space are the preconditions of perception. As such, they cannot themselves be given in perception. In short, Gross argued that Kant’s arguments are insufficient to establish time and space as pure a priori forms of intuition (1910, p. 19). In other words, Kant did not demonstrate that time and space are the necessary a priori preconditions of perception that do not themselves derive from perception. For example, the argument that time is the precondition without which no events could take place temporally does not achieve what Kant would like it to. Gross argued that this only proves that time must precede other perceptions, not that time itself could not be derived from sensory experience as Kant would have it (1910, p. 28–32). So Kant’s argument does not achieve what the philosopher wanted it to, namely to prove that time is a pure a priori form of intuition.

Contrary to Kant, Gross argued that time and space do, in fact, hail from perception. Kant did not realise this possibility, because he likely limited himself to the traditional five senses (Gross, 1910, p. 16). This means Kant overlooked a different kind of sensory experience, which is called *Vitalempfindung* in German (Gross, 1910, p. 16). An example of such a *Vitalempfindung* is the sensation of warmth and cold. The idea is that we experience warmth and cold not through the five senses but through some other organs. Another such *Vitalempfindung*, Gross argued, is the sensation of spatial awareness when we move (1910, p. 16). Similarly, our experience of time is also a *Vitalempfindung*. According to Gross, we become aware of time through our mental activity (1910, p. 30–31). His argument is that we are aware of time’s passing independently of any events taking place in it. This is because our mental activity brings forth time sensations [*Zeitempfindungen*]. Gross refers to this mental activity as attention [*Aufmerksamkeit*] and tension [*Spannung*] (Gross, 1910, pp. 40–41).

Gross concluded that time and space can be regarded as a priori forms of intuition, but not *pure* a priori forms (1910, p. 35). In other words, Kant was right in proposing that time and space are the a priori conditions for sensation, as long as “a priori” means “preceding all experience of external objects” (Gross, 1910, p. 28). However, time and space are not *pure* a priori forms, if “pure” is meant to imply the non-perceptual origins of those forms. That is because time and space are given through *Vitalempfindungen* (Gross, 1910, 20–21). Despite this, time and space are still the backdrop against which all other sensations take place. This can be explained by the fact that the organs which allow us to experience time and space are almost always affected (Gross, 1910, pp. 40–41). Our muscles, for example, are always active and thus supply us continuously with space-sensations. Since these are so constant, they naturally form a stable background against which all other sensations (the sensations of colours, smells, sounds, etc.) can be experienced.

It is clear that Uexküll and Gross have a similar programme, namely a critical engagement with and naturalisation of Kant’s transcendental idealism. For Uexküll too, time and space are a priori forms, in the sense that they underly all other sensory experience. But again, they are not *pure* forms of intuition, since they themselves are also derived from perception. Although the details differ, Uexküll and Gross argued that the concrete organisation of the organism shapes its time and space. For example, both authors emphasise the central role a subject’s muscles play in perception of space. The naturalisation of Kant’s pure forms is therefore clearly not unique to Uexküll.

In fact, there are strong reasons to suspect Uexküll’s views of time and space were inspired by Gross’s essay. For example, although Uexküll does briefly reflect on Kant’s ideas on time and space in his 1902 paper *Psychologie und Biologie in ihrer Stellung zur Tierseele* (pp. 215–216), he does not yet undertake a naturalisation of those ideas. In his 1909 work *Umwelt und Innenwelt der Tiere*, Uexküll does not explicitly connect his own project to Kant’s philosophy.^19^ It is thus very plausible that Gross’s naturalisation of Kant’s forms of intuition led Uexküll to develop a similar project. The close relation between Gross and Uexküll becomes even more apparent when one takes into account that in 1913, Uexküll published a research paper on crayfish in collaboration with Gross (1913). In the same year, Gross wrote an introduction to Uexküll’s *Bausteine zu einer biologischen Weltanschauung* (Gross, 1913, pp. 9–13). This close professional relationship renders Gross’s influence on Uexküll all the more plausible.

Nevertheless, there are significant differences between their respective projects. Firstly, Uexküll did not explicitly identify an organ or mental faculty that brings about time. Gross, as discussed, did identify mental activity as that which creates time sensations. Secondly, Gross and Uexküll disagreed on the origin of space. Uexküll had localised the origin of space in very specific organs, specifically in the muscles, nerve endings (including the cones and rods in the eye), and the semicircular canals.^20^ Gross, however, argued that our sense of space is the result only the motion of whole body parts (1910, p. 50). Nerve endings and irregular motions of muscles have no relevance for the creation of space (Gross, 1910, p. 59). Furthermore, Cyon was wrong in affirming that this organ creates our sense of space (Gross, 1910, p. 59). This organ does indeed allow us to navigate space, but only because a primordial representation [*Urvorstellung*] of space is already in place (Gross, 1910, pp. 59–60).

Another, albeit less consequential, difference is that Gross argued that time and space are continuous, whereas according to Uexküll, they are not. As previously discussed, Uexküll held that time and space are made up of their respective smallest bits, namely the moments and places. So although time and space may appear to be continuous, they are in fact discontinuous. Gross, however, argued that since the organs or mental processes from which our sense of space and time originate are always active, time and space themselves must be continuous (1910, p. 66).

### Uexküll and Gross on apperception and time

Gross is credited by Uexküll with demonstrating the relation between time and apperception (1928, p. 44). Clearly then, Gross influenced Uexküll’s work on this matter. To understand this, we must first investigate what Uexküll understood by apperception and how he considered its relation to time. It appears that he did not apply the concept in the same way as Kant did. As discussed previously, Kant distinguishes between many different kinds and functions of apperception. Uexküll, however, does not make such distinctions explicitly. Consequently, the exact meaning of apperception varies throughout his *Theoretische Biologie*. It will be useful, then, to attempt to disentangle the different uses and to see how they relate to Kant’s work on apperception. According to Uexküll, apperception is closely related to time: “Apperception is a life process, which proceeds in phases, in which each phase makes itself known through a sensible sign; this sign is the moment” (1928, p. 44).^21^

Uexküll goes on to expand on the relation between the moment-signs and apperception:

According to Kant, the unity of apperception creates the unity of our I, which is always supplied with a moment-sign, while it [the I] lacks a local-sign. Consequently, all psychical processes, feelings, and thoughts are always tied to a specific moment and proceed in the same time as the objective sensations. Time includes the subjective and the objective world in the same way, and does not make a distinction between them like space does. (1928, p. 45)^22^

In other words, the I that is created by the unity of apperception must always be combined with a moment-sign. After all, all mental events like thoughts and feelings take place in time, just like objective events. For example, I might experience the subjective sensation of pain and at the same time experience the objective sensation of seeing a knife cut my finger. Time, then, does not distinguish between inner and outer sensations, whereas space does. After all, mental events like thoughts do not have a local-sign and hence do not take place anywhere like an objective event does.

In this short passage, Uexküll covers a lot of ground. First of all, he refers to the unity of apperception and hence indirectly to transcendental apperception. The synthetic unity of apperception, as the possibility of synthesis, is a feature of transcendental apperception, which is the precondition for any kind of knowledge whatsoever. However, Uexküll also emphasises the empirical apperception, namely apprehension of one’s mental content. It has to be the case that the I always has a moment-sign, since all of my mental contents I apperceive also take place in time.

Apperception and time are connected in other ways as well. Time can be measured because of a regular change in the process of apperception: “In the process of apperception, certain moment-signs are specifically marked. The ability to note specific moment-signs is called attention” (Uexküll, 1928, p. 48).^23^ When we measure time, we observe the moment-signs through apperception. Some of these moment-signs will be marked with a specific quality, like the ticking of a metronome. This then allows us to measure time. One might, for example, say that it took five ticks for some motion to complete.

Later on, Uexküll wrote that perception time underlies everything in apperception (1928, p. 55). Presumably, he meant again that all of our mental states are temporally ordered and hence that everything we apperceive is also ordered likewise. Since apperception here is taken to mean apprehension of one’s mental content, its use is at this point more akin to empirical apperception than transcendental apperception.

At another point, Uexküll refers to the process of apperception [*Apperzeptionsprozeß*] as the process that synthesises objects (1928, p. 67). In other words, Uexküll likely refers to the synthetic unity of apperception. Besides these uses, Uexküll also applies the concept of apperception in ways that are more particular to his philosophical system. Uexküll wrote that there is a unity “which goes beyond even our own apperception, in which we would otherwise recognise the last unity” (1928, p. 70).^24^ There is yet a bigger unity than the unity of apperception, namely some kind of vital force. Hence, there is some kind of precondition for knowledge that even precedes the transcendental apperception. Of course, we cannot know this power directly. Nevertheless, we can use our apperception to construct certain rules to approximate the immaterial force that constructs nature in orderly fashion (Uexküll, 1928, p. 144).

Uexküll credited Gross with demonstrating the close relationship between time and apperception (1928, p. 44). As explained in § 4.1, Gross had argued that time is not, as Kant would have it, a *pure* form. Rather, time itself is firstly given in perception and does not precede it. Therefore, Gross has to solve the following problem: If time itself is an impression, how is it possible that it is separated from and becomes the background for other sense impressions (Gross, 1910, p. 61)? In other words, how can something that is sensible be a form of intuition?

To answer this question, Gross explicitly refers to Kant’s concept of the understanding [*Verstand*] (1910, p. 62–64). Kant defined the understanding as follows: “[T]he understanding … is our faculty of producing representations by ourselves, or the spontaneity of knowledge” (B76). The understanding, as the active faculty that produces representations, creates time by what Gross called an act of apperception [*Apperzeptionstätigkeit*]:

We have previously defined [time sensations] as immediate sensations of a … psychical activity of the conscious I. Now we know also which activity. It is the act of apperception of the understanding. Each perception and feeling is connected into a single intuition [*Anschauung*] by the understanding’s apperception; the perceiving of this act of apperception is time. (1910, p. 64)^25^

As seen in § 4.2, Gross defined time as the result of some mental activity he called attention. Now, it appears that this mental activity is nothing less than our awareness of the transcendental apperception itself (1910, p. 65).

The question of how time is separate from other sense impressions still needs to be answered. The argument goes as follows. There is a kind of psychological activity which produces time sensations (Gross, 1910, p. 66). One might call this activity transcendental apperception, attention, or spontaneity, since Gross considered these concepts to be roughly synonymous. Since this psychological activity is always active, the time sensations which it produces are also always present in our consciousness. These then naturally become one continuous intuition of time [*Anschauung*]. Other sensations are also psychological events. Hence, they also bring forth time sensations and are therefore themselves temporal. But time is continuously present in our consciousness even without any other sensations whatsoever. Time thus exists independently of other sensations. That is how time becomes a form of intuition that is prior to other sensations despite being a sensation itself.

Uexküll’s and Gross’s views on the relation between time and apperception are similar but differ in one major way. They agree that apperception synthesises time and sensations into our experience. The main disagreement appears to be that for Gross, apperception is in fact the activity that creates time. Uexküll, however, remained agnostic about the origin of our time sensations. Time is of course related to apperception and the *Gemüt*. Perhaps he meant to suggest the same point as Gross, namely that apperception creates our time sensation. But this argument is never explicitly made in his text. It is the case, however, that a certain mental act allows us to measure time. As discussed, we can measure time because a mental faculty allows us to introspect on the apperception process. Uexküll referred to this faculty as attention, which Gross used as a rough synonym for apperception.

## Conclusion

To conclude, Uexküll sought to establish that time and space are products of the biological constitution of the subject. Time and space are dependent on the subject, and objective time and space are merely useful conventions. Rather, they are foundational properties of the subject’s *Umwelt*. Much like Kant referred to time and space as the pure form of intuition, Uexküll saw this conceptual pair as fundamental constituents of the subject’s world. The concept of apperception further demonstrates Uexküll’s indebtedness to Kant. Gross and Uexküll both used this concept rather imprecisely. Generally, they used apperception as a catch-all term for the synthetic powers of the subject as well as certain mental faculties like attention. Although the exact function of apperception remains ambiguous, it is clear that it is closely related to time for Uexküll. Uexküll’s discussion of time and space is therefore primarily rooted in Kantian philosophy.

However, on the basis of the works of Gross and empirical research of his time, Uexküll in effect naturalised Kant’s pure forms of intuition. This is especially evident in the case of space. Uexküll identified certain organs (nerves, muscles, the semicircular canals) as the originating factors of the subject’s space. In the case of time, Uexküll did not identify any organs that might be capable of producing that particular foundational property. Nevertheless, Uexküll argued that one could ascertain facts about another subject’s time through empirical research and held that the subject’s time is always suited perfectly for its biological needs.

This naturalisation must not be understood as an attempt to fit Kantian philosophy into a physicalist mould. Rather, Uexküll combined his Kantian background with his own specific brand of vitalism. This has led to continuous tensions in the biologist’s own work between Kantian transcendental idealism on the one hand, and vitalism and empirical science on the other.

Uexküll’s project is unique in the sense that he developed his own metaphysical system, but the naturalisation of Kant’s pure forms was not unique. Particularly striking are the similarities between Gross’s and Uexküll’s accounts of time and space. Gross, too, drew on contemporary empirical research to formulate his own naturalised account of Kant’s pure forms of intuition. Gross’s text may be little-known, but it preceded and clearly influenced Uexküll’s. Furthermore, the fact that both Gross and Uexküll drew on a myriad of authors for their naturalised Kantianism shows that further research on the naturalisation of Kant’s pure forms of intuition and related debates about time and space is needed.

An important area of investigation in this regard will be the works of the Kant-influenced scientists Johannes Müller (1801–1858) and Hermann Helmholtz (1821–1894), whose works on physiology and perception paved the way for the naturalisation of Kant’s works (Finger and Wade 2002a, b; see also Turner, 1994). But many more authors, like Karl-Ernst von Baer and Elias von Cyon as mentioned in this article, were concerned with Kantian philosophy in its relation to the life sciences. The naturalisation of Kant in the life sciences of the 19th and early 20th century will therefore prove to be fertile ground for further research.

## Data Availability

Not applicable.

## Footnotes

1 For an overview of this reception, see the seventh chapter of Carlo Brentari’s monograph (2015).

2 See Kull (2001) and Kull (2020).

3 That is of course not to say that it has received no attention at all. See the aforementioned work by Brentari (2015), Esposito (2020), and Heredia (2020). Mildenberger’s 2007 biography of Uexküll is of course also relevant in this regard.

4 For more on this, see Brentari (2015) and Kull (2001).

5 The translations of *Merkwelt* and *Wirkwelt* are taken from Joseph D. O’Neill’s 2010 translation of Uexküll’s *Streifzüge durch die Umwelten von Tieren und Menschen*.

6 For Uexküll’s *Theoretische Biologie*, I use the second edition. As far as I am aware, there is no English translation of the second edition. Therefore, all translations from that particular work are mine. For translations of *Streifzüge*, I use the 2010 translation by O’Neill.

7 References to Kant’s *Critique of Pure Reason* make use of the page numbers of the first A-edition or the second B-edition. All are taken from the 2007 Penguin Books edition of that work.

8 I will be limiting myself to Uexküll’s views on the subject’s time experience. For Uexküll on ontogenetic time, see Magnus (2011).

9 Rama notes that the Baltic-German biologist Karl Ernst von Baer’s use of musical metaphors influenced Uexküll in this regard (2024, pp. 379). I suspect, however, that the connection made here by Uexküll between time, number, and musicality is inspired by the work of the Russian-French physiologist Elias von Cyon. Von Cyon argued for a close connection between the perception of time, space, mathematics, and musicality (1907, pp. 531–532).

10 I owe the translation of *Merkzeit* as perception time to O’Neill’s 2010 translation of *Streifzüge*.

11 Magnus notes that one colleague of Uexküll, Gerhard Brecher, argued that some activity in the nervous system is responsible for bringing forth time-sensations (2011, p. 47). In short, Brecher argued that the moment, as smallest unit of time, is a product of the nervous system (Brecher, 1932). But it is not obvious that Uexküll accepted this conclusion in his *Theoretische Biologie*.

12 Kant also frequently uses the concept of *Gemüt*. In his commentary on Samuel Thomas von Sömmerring’s *Über das Organ der Seele*, Kant defines it as the faculty that that unites representations and which effects the unity of empirical apperception (Kant, 1796, p. 83).

13 At this point, one might be reminded of the work of the philosopher Arthur Schopenhauer, who was also greatly influenced by Kant. In brief, Schopenhauer argued that we could, in fact, know the thing-in-itself. He identified this thing-in-itself with a blind and eternal striving power, which he called the will. For an introduction to Schopenhauer and relevant literature, see Wicks (2024). However, Schopenhauer’s influence on Uexküll is difficult to establish with certainty. To my knowledge, Uexküll never explicitly mentions Schopenhauer. It is, of course, possible some ideas from Schopenhauer’s work came to Uexküll through the work of other authors. The German-American physiologist and biologist Jacques Loeb, for example, mentions Schopenhauer as an inspiration for his own analysis of will (1912, p. 35). Uexküll engaged with Loeb’s work on tropisms in his *Theoretische Biologie* (1928, pp. 223–225). Perhaps, then, Schopenhauer’s ideas entered into Uexküll’s works via Loeb. But this connection needs further research.

14 My translation. “Hier stoßen wir hart auf hart mit einem in uns auftretenden Naturfaktor zusammen, der, obgleich er in die Tätigkeit unseres Gemütes planmäßig eingefügt ist, dennoch unerkennbar bleibt … Aus einer uns unzugänglichen Ursprungsstätte rollt sich die zeitlich gegliederte Kette von Willensdirektiven ab” (Uexküll, 1928, p. 22)

15 My translation. “Es wird sich möglicherweise dabei herausstellen, daß die gesamte Zeitspanne des Nu auf die Muskeltätigkeit anzurechnen ist und nichts für den Willensimpuls übrigbleibt” (Uexküll, 1928, p. 56).

16 Uexküll’s vitalistic thing-in-itself which organises monadic subjects also evokes Platonic and Leibnizian philosophy respectively. A thorough examination of this background is however beyond the scope of this paper. For a discussion on this subject, see Brentari (2020).

17 See also *The Semicircular Canals and the “Sense of Space”* (1878) for a brief summary of these points.

18 See also *Atlas zur Bestimmung der Orte der Tiere* (1927), in which Uexküll and Brock do exactly that.

19 Uexküll does, however, refer to Kant in a short text titled *Gedanken über die Entstehung des Raumes* (Uexküll, 2006, p. 85). This text was originally published in 1913, three years after Gross’s work.

20 The expression “origin of space” here and similar expressions elsewhere in this text are somewhat cumbersome. However, they are necessary in order to emphasise that Uexküll held that time and space are produced by the subject and that he was not just writing about the subject’s *experience* of time and space.

21 My translation. “Die Apperzeption ist ein Lebensprozeß, der in Phasen vor sich geht, wobei jede Phase sich durch ein sinnliches Zeichen kund gibt: dieses Zeichen ist der Moment” (Uexküll, 1928, p.44).

22 My translation. “Nach Kant erzeugt die Einheit der Apperzeption die Einheit unseres Ich, das stets mit einem Momentzeichen versehen ist, während ihm ein Lokalzeichen mangelt. Infolgedessen sind alle psychischen Vorgänge, Gefühle und Gedanken stets and einen bestimmten Moment gebunden und verlaufen in der gleichen Zeit wir [sic.] die objektiven Empfindungen. Die Zeit umfaßt in gleicher Weise die subjektive wie die objektive Welt und macht keinen Unterschied zwischen ihnen wie der Raum” (Uexküll, 1928, p. 45).

23 My translation, “Es werden … beim Ablauf der Apperzeption bestimmte Momentzeichen besonders angemerkt. Die Fähigkeit, bestimmte Momentzeichen besonders zu bemerken, bezeichnen wir als Aufmerksamkeit.” (Uexküll, 1928, p. 48).

24 My translation. “Und daß wir hier auf dem Wege sind, einem Naturwalten nachzuspüren, das auf eine Einheit hinweist, die noch über unserer eigenen Apperzeption steht, in der wir sonst die letzte Einheit zu erkennen vermögen” (Uexküll, 1928, p. 70).

25 My translation. “Wir definierten sie oben als unmittelbare Empfindungen einer physischen und psychischen Tätigkeit des bewußten Ich. Jetzt wissen wir auch welcher Tätigkeit. Es ist die Apperzeptionstätigkeit des Verstandes. Alle Empfindungen und Gefühle werden vom Verstande durch seine Apperzeption zu einer Anschauung verbunden: das Empfinden dieser Apperzeptionstätigkeit ist die Zeit” (Gross, 1910, p. 64).

## References

[CR2] Brecher, G. A. (1932). Die Entstehung und Biologische Bedeutung der subjektiven Zeiteinheit, - Des Momentes. *Zeitschrift für vergleichende Physiologie*, *18*, 204–243. 10.1007/BF00338160

[CR3] Gross, F. (1910). *Form und Materie des Erkennens in der transzendentalen Ästhetik. Eine erkenntnistheoretische Untersuchung*. Verlag von Johann Ambrosius Barth.

[CR4] Gross, F. (1913). Einleitung: Biologie als Weltanschauung. In J. Uexküll (Ed.), *Bausteine zu einer biologischen Weltanschauung: Gesammelte Aufsätze* (pp. 9–13). F. Bruckmann.

[CR5] Kant, I. (1796). In S. T. von Sömmerring. *Über das Organ der Seele*. Friedrich Nicolovius.

[CR6] Kant, I. (2007). *Critique of pure reason* (M. Weigelt, Trans. & Ed.). Penguin Books. (Original work published 1787).

[CR7] Loeb, J. (1912). *The mechanistic conception of life*. University of Chicago Press.

[CR16] von Cyon, E. (1907). Das Ohrlabyrinth als Organ der mathematischen Sinne für Raum, Zeit und Zahl. *Archiv für die gesamte Physiologie des Menschen und der Tiere*, *118*, 525–535. 10.1007/BF01677511

[CR17] von Cyon, E. (1908). *Das Ohrlabyrinth als Organ der mathematischen Sinne für Raum und Zeit*. Springer.

[CR8] von Uexküll, J. (1902). Psychologie und Biologie in ihrer Stellung zur Tierseele. *Ergebnisse der Physiologie*, *1*, 212–233. 10.1007/BF02320908

[CR9] von Uexküll, J. (1909). *Umwelt und Innenwelt der Tiere*. Springer.

[CR11] von Uexküll, J. (1928). *Theoretische Biologie* (2nd edition). Springer.

[CR10] von Uexküll, J. (2006). Gedanken über die Entstehung des Raumes. In J. Dünne, & S. Günzel (Eds.), *Raumtheorie. Grundlagentexte aus Philosophie und Kulturwissenschaften* (pp. 85–94). Suhrkamp. (Original work published 1913).

[CR13] von Uexküll, J., & Brock, F. (1927). Atlas zur Bestimmung der Orte in den Sehräumen der Tiere. *Zeitschrift für vergleichende Physiologie*, *5*, 167–183. 10.1007/BF00340818

[CR14] von Uexküll, J., & Gross, F. (1913). Studien über den Tonus. VII. Die Schere des Flußkrebses. *Zeitschrift für Biologie*, *60*(8–9), 334–357.

[CR15] von Uexküll, J., & Kriszat, G. (1956). *Streifzüge durch die Umwelten von Tieren und Menschen* und *Bedeutungslehre*. Rowohlt. (Original works published 1934 & 1940).

[CR12] von Uexküll, J. (2010). *A foray into the worlds of animals and humans*, with *A theory of meaning*. (J. D. O’Neill, Trans.). University of Minnesota Press. (Original works published 1934 & 1940).

[CR19] Allison, H. E. (2004). *Kant’s transcendental idealism. An interpretation and defense* (2nd edition). Yale University Press.

[CR20] Brentari, C. (2015). *Jakob von Uexküll: The discovery of the umwelt between biosemiotics and theoretical biology*. Springer.

[CR21] Brentari, C. (2020). Kantian Monads in a Platonic World. Some Remarks on the Philosophical Background of Jakob von Uexküll’s *Umweltlehre*. *Thaumàzein*, *8*, 246–259. 10.13136/thau.v8i

[CR22] Engstrom, S. (2013). Unity of apperception. *Studi Kantiani*, *26*, 37–54.

[CR23] Esposito, M. (2020). Kantian ticks, Uexküllian melodies, and the transformation of transcendental philosophy. In F. Michelini, & K. Köchy (Eds.), *Jakob von Uexküll and philosophy: Life, environments, anthropology* (pp. 36–51). Routledge.

[CR37] Finger, S., & Wade, N. J. (2002a). The neuroscience of Helmholtz and the theories of Johannes Müller. Part 1: Nerve cell structure, vitalism, and the nerve impulse. *Journal of the History of the Neurosciences, 11*(2), 136–155. 10.1076/jhin.11.2.136.15190 10.1076/jhin.11.2.136.15190 12122806

[CR38] Finger, S., & Wade, N. J. (2002b). The neuroscience of Helmholtz and the theories of Johannes Müller. Part 2: Sensation and perception. *Journal of the History of the Neurosciences, 11*(3), 234–254. 10.1076/jhin.11.3.234.10392 10.1076/jhin.11.3.234.10392 12481475

[CR26] Heredia, J. M. (2020). Jakob von Uexküll, an intellectual history. In F. Michelini, & K. Köchy (Eds.), *Jakob von Uexküll and philosophy: Life, environments, anthropology* (pp. 17–35). Routledge.

[CR27] Kull, K. (2001). Jakob von Uexküll: An introduction. *Semiotica*, *134*, 1–59.

[CR28] Kull, K. (2020). Uexküll studies after 2001. *Sign System Studies*, *48*(2–4), 483–509. 10.12697//SSS.2020.48.2-4.13

[CR29] Magnus, R. (2011). Time-plans of the organisms: Jakob von Uexküll’s explorations into the temporal constitution of living beings. *Sign System Studies*, *39*, 37–56.

[CR30] Magnus, R., & Kull, K. (2009). Exemplifying *Umweltlehre* through one’s own life: A biography of Jakob von Uexküll by Florian Mildenberger. Review of *Umwelt als Vision: Leben und Werk Jakob von Uexkülls* (1864–1944). By Florian Mildenberger Stuttgart, Franz Steiner Verlag, 2007, 320 pages. *Biosemiotics*, 2, 121–125.

[CR31] Mildenberger, F. (2007). *Umwelt als Vision: Leben und Werk Jakob von Uexkülls*. Franz Steiner. 18074886

[CR40] Rama, T. (2024). The explanatory role of *Umwelt* in evolutionary theory: Introducing von Baer’s reflections on teleological development. *Biosemiotics, 17*, 361-386. 10.1007/s12304-024-09569-8

[CR33] The Semicircular Canals and the Sense of Space. (1878). *Mind*, 3(12), 559–564.

[CR34] Turner, R. S. (1994). *In the eye’s mind. Vision and the Helmholtz-Hering controversy*. Princeton University Press.

[CR35] Vailati, E. (1998). Introduction. In S. Clarke (Ed.), *A demonstration of the being and attributes of God and other writings* (pp. ix–xxxi). Cambridge University Press.

[CR36] Wicks, R. (2024). Arthur Schopenhauer. In *The Stanford **encyclopedia of **philosophy*. Retrieved February 16, 2025. https://plato.stanford.edu/entries/schopenhauer/#4

